# Efficacy of Faricimab in the Treatment of Diabetic Macular Edema and Faricimab-Related Changes in OCT and OCT Angiography

**DOI:** 10.3390/pharmaceutics17070858

**Published:** 2025-06-30

**Authors:** Dorota Śpiewak, Łukasz Drzyzga, Mariola Dorecka, Katarzyna Witek, Dorota Wyględowska-Promieńska

**Affiliations:** 1 Department of Ophthalmology, Prof. K. Gibiński University Clinical Center, Medical University of Silesia, 40-514 Katowice, Poland; 2 Clinical Ophthalmology Center Okolux, 40-754 Katowice, Poland; 3 Department of Ophthalmology, Faculty of Medical Sciences in Katowice, Medical University of Silesia, 40-287 Katowice, Poland

**Keywords:** faricimab, anty-VEGF, diabetic macular edema, optical coherence tomography, optical coherence tomography angiography

## Abstract

Our study aimed to assess the anatomical changes in the retina, including the assessment of the reduction of diabetic macular edema (DME) on optical coherence tomography (OCT) and the improvement of retinal microvascular parameters, defined by the reduction of nonperfusion areas on OCT angiography (OCTA) after intravitreal injections of 6 mg faricimab, an anti-VEGF drug used in the treatment of DME. The study included twenty-two patients aged between 61 and 74 years, each of whom received four loading doses of 6 mg faricimab at 1-month intervals, as described in the summary of product characteristics. Hemodynamic parameters were analyzed by OCT angiography before the first intravitreal injection of faricimab and one month after each subsequent injection. The following parameters were analyzed: non-perfusion area (NPA), superficial capillary plexus (SCP) and deep capillary plexus (DCP), outer retinal flow area (ORFA), choriocapillaris flow area (CCFA) and foveal avascular zone (FAZ). Despite differences in the magnitude of improvement and time to improvement from the start of treatment with intravitreal injections of 6 mg faricimab, reductions in DME and improvements in OCTA parameters resulted in increased retinal blood flow and better visual acuity.

## 1. Introduction

Diabetic retinopathy (DR) is the leading cause of impaired visual acuity and blindness in highly developed countries. It is estimated that the number of people worldwide with diabetes mellitus (DM) will increase from 415 million in 2015 to 642 million by 2040 [1]. It has also been shown that women with type 2 DM have a higher prevalence of diabetic retinopathy than men. However, men exhibit more severe forms of retinopathy, worse visual acuity or even blindness [2]. DR is a common microvascular complication of diabetes. Hyperglycaemia and altered metabolic pathways lead to oxidative stress, inflammatory processes, overexpression of vascular endothelial growth factor (VEGF), and the development of neurodegeneration observed in diabetic retinopathy [3]. Therefore, regular eye check-ups and close interdisciplinary cooperation are crucial in preventing vision loss [4]. Improvements in best-corrected visual acuity (BCVA) and a reduction in central retinal thickness (CRT) have been well-documented after intravitreal injections of anti-VEGF drugs in patients with diabetic macular oedema (DME). However, the effects of these drugs on macular microcirculation in diabetic retinopathy still need to be investigated.

The pathomechanism of DR is complex and associated with chronic hyperglycemia, hypoinsulinemia, dyslipidemia and hypertension. These systemic abnormalities induce oxidative stress, formation of advanced end-products of glycation, microvascular changes, inflammation and neurodegeneration of the retina [2]. DR and DME result from inflammatory processes and VEGF overexpression, primarily due to retinal ischaemia and hypoxia.

Anti-VEGF drugs play a key role in the treatment of diabetic macular edema. VEGF mediate cellular responses initiated by VEGF binding to tyrosine kinase receptors (VEGFRs) on the cell surface, resulting in the dimerization of the receptor and subsequent activation and transphosphorylation of tyrosine kinase [5]. Angiogenic factors bind to their receptors, activating endothelial cells, which are usually resistant to neovascular stimuli. In particular, VEGF-A, the primary factor for angiogenesis, and placental growth factor (PLGF) have been shown to activate quiescent endothelial cells and promote their proliferation, migration, and increased permeability [5,6]. The angiopoietin/Tie signaling pathway is another essential system in vascular development, remodeling and angiogenesis, typical of retinal vascular diseases. Angiopoetin-1 (Ang-1) and angiopoetin-2 (Ang-2) are crucial regulators of vascular stability. Ang-1 and 2 are ligands for the receptor tyrosine kinase Tie2, found mainly in vascular endothelial cells. Their roles in angiogenesis are essentially opposite. Ang-1 acts as a receptor activator and induces Tie2 signaling, thus promoting vascular stability. Ang-2, on the other hand, can act as an antagonist that blocks Tie2 signaling, leading to the development of pathological conditions. The action of Ang-2 appears to be VEGF-dependent. In the absence of VEGF, Ang-2 promotes vessel regression; the presence of VEGF induces angiogenesis [5].

In ischaemic diseases, such as diabetic retinopathy, Ang-2 becomes upregulated. Consequently, Tie-2 is inactivated, triggering vascular leakage, pericyte loss and inflammation. Recombinant Ang-1, combined with Ang-2 blocking molecules and vascular endothelial tyrosine phosphatase inhibitors (VE-PTP), reduce vascular leakage, thus showing therapeutic effects in diabetes, atherosclerosis and ocular neovascular diseases. In addition, recent studies have shown that angiopoietin-like proteins may play an essential role in cellular metabolism, leading to retinal vascular disease. Furthermore, some new research demonstrates that angiopoietin-like proteins may play a prime role in cellular metabolism, leading to retinal vascular disease. Faricimab has a dual mechanism of action; it binds and neutralizes the VEGF and Ang-2 pathways and, therefore, may demonstrate superior efficacy compared to other anti-VEGF drugs. For this reason, faricimab may significantly improve visual acuity in patients with DME [7].

DME can develop at any stage of DR, regardless of its severity, in both non-proliferative diabetic retinopathy (NDPR) and its proliferative form (PDR) [8]. One of the causes of fluid accumulation within the macula is retinal ischemia. Fluid accumulation under ischemic conditions is believed to result from damage to endothelial cells, disruption of the tight junctions of the blood-retinal barrier and consequent excessive vascular permeability, which is also increased by the coexisting inflammatory processes [9]. Damage to the blood-retinal barrier primarily leads to the development of DME. At the same time, leakage of pro-inflammatory cytokines and plasma proteins into the extra-capillary space triggers the formation of hard exudates. As the disease progresses, vasoconstriction and capillary obstruction lead to capillary damage and retinal ischemia. At this stage of the disease, so-called cotton wool spots can be observed on fundus examination [3]. Based on the clinical manifestations, a classification of NPDR has been established. In the mild form, the vascular endothelium is damaged, and microaneurysms develop. In the moderate form, in addition to microaneurysms, hard exudates, cotton wool balls, and macular intraretinal haemorrhages appear. The severe form of NPDR, according to the American Academy of Ophthalmology (AAO), is defined by the 4:2:1 rule, which consists of the presence of intraretinal hemorrhages in all quadrants, venous beading in at least two quadrants, or intraretinal microvascular abnormalities (IRMAs) in at least one quadrant. In the final stage of DR, severe hypoxia leads to the development of proliferative diabetic retinopathy, associated with neovascularization, including neovascularization of the disc (NVD), neovascularization elsewhere (NVE) and neovascularization of the iris (NVI). It also causes vitreoretinal proliferation, tractional retinal detachment, hemorrhages into the vitreous, and neovascular glaucoma [3,10].

An unsatisfactory response to the anti-VEGF regimen has been observed in some patients with DME, suggesting that the concomitant occurrence of edema and diabetic macular ischemia (DMI) may reduce the response to treatment [9].

The advent of anti-VEGF drugs marks a breakthrough in treating retinal vascular diseases. The medications approved by the Food and Drug Administration (FDA) and the European Medicines Agency (EMA) for DME treatment are ranibizumab, aflibercept (doses of 2 mg and 8 mg), and faricimab. In our observation, patients were treated with faricimab.

Faricimab is the first bispecific monoclonal intraocular antibody targeting two distinct receptor pathways, i.e., it binds to and neutralizes Ang-2 and VEGF-A. Ang-2 inhibition contributes to blood vessel stability by reducing vascular leakage, alleviating neovascularization and inhibiting inflammatory processes. An analysis of population pharmacokinetics revealed that the maximum concentrations of free (unbound to VEGF-A and Ang-2) faricimab in the plasma (Cmax) occur approximately two days after administration. The mean (+/−SD) plasma Cmax were 0.23 (0.07) µg/mL and 0.22 (0.07) µg/mL in nAMD and DME patients, respectively. After repeated intravitreal injections, the mean plasma concentrations of faricimab are expected to be 0.002–0.003 µg/mL with injections every 8 weeks. Desideri et al. demonstrated dose-proportional pharmacokinetics (based on Cmax and AUC) of faricimab at a dose range of 0.5–6 mg. After monthly intravitreal injections, no accumulation of faricimab was found in the vitreous humor or the plasma [11]. Maximum concentrations of free faricimab in the plasma are estimated to be approximately 600 and 6000-fold lower than in the aqueous humor and body, respectively. Therefore, systemic pharmacodynamic effects are unlikely as no significant changes in plasma free-VEGF and Ang-2 concentrations during faricimab treatment were observed in clinical trials [5,11,12].

## 2. Material and Methods

### 2.1. Intravitreal Drug Delivery

Twenty eyes with DME were treated with four intravitreal injections of 6 mg faricimab (Vabysmo, Roche Pharma AG, Grenzach-Wyhlen, Germany) at monthly intervals (8 female and 12 male patients, aged between 61 and 74 years, all diagnosed with diabetic macular edema). Each injection contained 6 mg of faricimab (0.05 mL solution). Injections were administered in the superior temporal quadrant, 3.5 mm from the limbus in pseudophakic eyes and 4 mm from the limbus in phakic eyes. Intravitreal injections were given by a single specialist in the operating room. All procedures were performed under aseptic conditions by the standard of care. Before and after the injection, 5% povidone-iodine solution (Betadine solution, Egis, France) was administered into the conjunctival sac.

The approval of the Bioethics Committee was obtained (BNW/NWN/0052/KB/128/25). All patients signed informed consent forms to receive intravitreal injections.

### 2.2. OCT and OCTA Scan Acquisition

Optical coherence tomography (OCT) and optical coherence tomography angiography (OCTA) were performed using Angiovue software for optical coherence tomography angiography (OCTA) Solix V1.1.0.022 Optovue Inc., Fremont, CA, USA—version: SOLIX FullRange AngioVue Expert.

### 2.3. OCT and OCTA Analysis

OCT and OCTA were performed before the first injection and one month after each subsequent administration of 6 mg faricimab.

OCT served to assess CRT (µm) in the central 1 mm circle of the ETDRS grid.

Superficial capillary plexus (SCP) and deep capillary plexus (DCP), foveal avascular zone (FAZ), outer retinal flow area (ORFA) and choriocapillaris flow area (CCFA) were measured automatically using OCTA software. Only non-perfusion areas (NPA) were measured manually but by a single examining ophthalmologist.

Automatic measurements of ORFA and CCFA allowed for the generation of vessel density maps covering a 6.4 × 6.4. mm area.

SCP and DCP vessel density was measured across the ETDRS grid with concentric circles at 1, 3, and 6 mm centred at the fovea.

## 3. Statistical Analysis

Statistical analyses were conducted using the R Statistical language (version 4.3.3; R Core Team, 2024) on Windows 11 Pro 64-bit. All analyses were performed using a two-sided alpha level of 0.05. As most clinical parameters deviated from a normal distribution, numerical variables were summarized using medians (*Mdn*) and interquartile ranges (*IQR*) to robustly represent central tendency and variability. The primary temporal effect was further adjusted for potential confounding by including sex and age as covariates. Statistical significance (*p*) and 95% confidence intervals (95% CIs) were derived using an asymptotic *t*-test approximation, ensuring robust inference for the estimated parameters.

## 4. Results

### 4.1. Baseline Characteristics of the Study Parameters

The efficacy of faricimab in treating diabetic macular edema was assessed using optical coherence tomography and optical coherence tomography angiography. The assessment was based on the dynamics of the CRT, NPAs, SCP and DCP vessel densities, ORFA, CCFA, FAZ and BCVA.

Most eyes exhibited moderate to advanced disease severity at presentation, showing a clear need for prompt and targeted therapeutic intervention to maintain or improve visual outcomes. Specifically, half of the eyes exhibited notable macular edema, as evidenced by a median central retinal thickness exceeding 500 µm. Non-perfusion areas of approximately 3 mm^2^ and reduced flow densities in the SCP and DCP indicated meaningful microvascular compromise. Increases in the choriocapillaris flow area, outer retinal flow area, and enlarged foveal avascular zone were consistent with overall retinal structural changes. Visual acuity at a median of 0.30 on the Snellen chart indicated moderate functional impairment.

### 4.2. Longitudinal Evaluation of CRT over Four Months of Faricimab Therapy

The linear mixed-effects model revealed substantial reductions in central retinal thickness compared to baseline, with highly significant improvements observed at one month (β = −115.86, *95% CI*: −143.36–−88.36, *p* < 0.001), two months (β = −147.83, *95% CI*: −175.33–−120.32, *p* < 0.001), three months (β = −175.76, *95% CI*: −203.26–−148.26, *p* < 0.001), and four months (β = −183.50, *95% CI*: −211.00–−156.00, *p* < 0.001).

The baseline intercept of approximately 496 µm (*95% CI*: 444–548) suggests that the study population, comprising older adults with diabetic macular edema, exhibited a markedly increased retinal thickness. Over four months, the cumulative effect size was close to an 184 µm decrease, exceeding thresholds considered relevant for meaningful clinical improvements in retinal pathology.

Neither patient sex (β = −38.00, *95% CI*: −103.58–27.59, *p* = 0.253) nor age (centred by the median of 66 years; β = −1.85, *95% CI*: −8.42–4.71, *p* = 0.576) exerted a statistically significant effect on treatment response, suggesting that the therapeutic impact of faricimab did not meaningfully vary across older demographic profiles.

**Figure 1:**
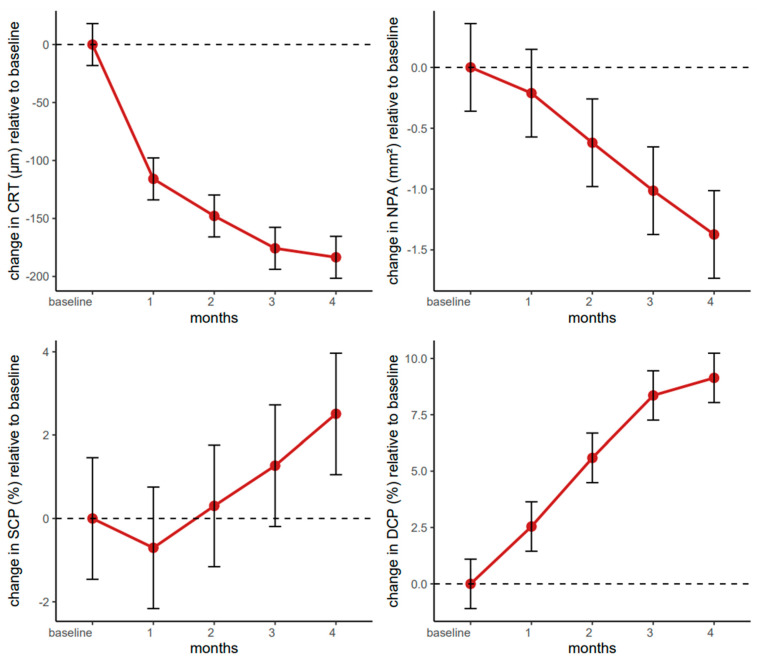
Changes in CRT (**upper left**), NPA (**upper right**), SCP (**lower left**) and DCP (**lower right**) relative to baseline over four months of faricimab therapy (the black dashed line represents the baseline level, the red solid line indicates changes in the studied parameters, and the black bars represent the 95% confidence intervals at specific time points).

Overall, the trajectory of CRT reduction emphasizes faricimab’s potency in alleviating retinal edema over a relatively short timeframe and highlights the therapy’s potential to provide consistent benefits in a heterogeneous population of older adults with diabetic macular edema (Figure 1).

**Figure 2:**
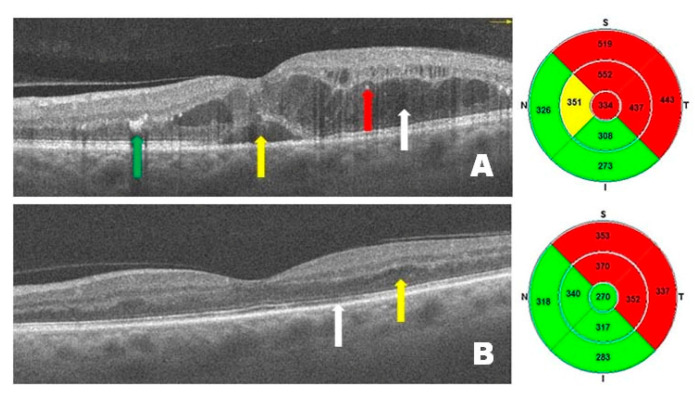
Patient 1—(**A**) Pre-treatment OCT scan: diffuse (white arrow) and serous (yellow arrow) edema, hard exudates (green arrow) and hyperreflective foci (red arrow) within the macula. (**B**) Post-treatment OCT scan: significant reduction in intraretinal and subretinal edema and the number of intraretinal cysts, slightly disturbed structure of the retina (yellow arrow), minor RPE and photoreceptor defects (white arrow).

Figure 2 presents OCT scans from a patient with DME before (Figure 2A) and after (Figure 2B) treatment with intravitreal injections of faricimab. Before treatment, intramacular and subretinal fluid accumulation is visible, causing diffuse and serous macular edema, with the presence of hard exudates and hyperreflective intramacular foci (HF). After treatment, a marked improvement is observed, accompanied by a reduction in central retinal thickness. DME has resolved—no intraretinal or subretinal hyporeflective spaces (under the neurosensory retina and above the retinal pigment epithelium) are present, and normal retinal anatomy (layers) is visible. These changes have resulted in significant improvements in visual acuity.

### 4.3. Longitudinal Evaluation of NPAs over Four Months of Faricimab Therapy

The linear mixed-effects model for NPAs indicated that faricimab produced meaningful decreases in NPAs, particularly from the second month onward. The estimated change (β = −0.21, *95% CI*: −0.57–0.14, *p* = 0.241) did not reach statistical significance one month post-baseline. However, the reductions became significant at two months (β = −0.62, *95% CI*: −0.98–−0.26, *p* = 0.001), three months (β = −1.01, *95% CI*: −1.37–−0.66, *p* < 0.001), and four months (β = −1.37, *95% CI*: −1.73–−1.02, *p* < 0.001).

The intercept of 2.25 (*95% CI*: 1.13–3.36, *p* < 0.001) suggests that patients started with relatively large NPAs at baseline, progressively decreasing over the four-month therapy period. Interestingly, sex emerged as a significant predictor, with male patients exhibiting a 2.34 mm^2^ larger NPA (*95% CI*: 0.93–3.75, *p* = 0.001) than female counterparts. Age, centred at 66 years, did not have a meaningful effect on these changes (β = −0.01, *95% CI*: −0.16–0.13, *p* = 0.836).

Overall, these findings support the effectiveness of faricimab in reducing non-perfused retinal areas in patients with diabetic macular edema, with a statistically robust effect detectable from the second month of therapy onward (Figure 1).

**Figure 3:**
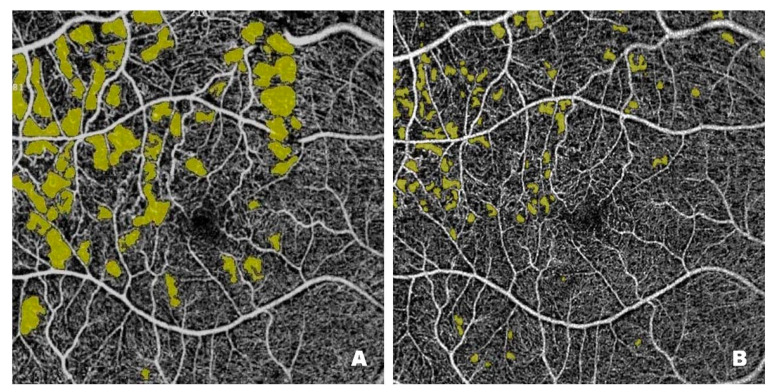
Patient 1—(**A**) Pre-treatment NPAs (3.898 mm^2^): regions lacking capillary flow, devoid of vascular pattern are marked in yellow; enlarging NPA areas connect with the FAZ zone. (**B**) Post-treatment NPA (1.430 mm^2^): a significant reduction in the yellow-marked areas, capillary circulation significantly restored.

Figure 3 shows NPAs on OCTA scans from a patient with DME before (Figure 3A) and after (Figure 3B) treatment with intravitreal injections of faricimab. Pre-treatment scans reveal numerous NPA foci measured in the superficial layers of the retina; the software sums their area and displays the result in mm^2^. In Optovue Solix, NPA areas are marked in yellow. After treatment, a clear improvement in capillary perfusion is seen as a reduction in the area of NPA zones marked in yellow. Improved retinal vascularization protects against the development of pathological retinal neovascularization with its numerous consequences, including blindness.

### 4.4. Longitudinal Evaluation of the SCP Vessel Density over Four Months of Faricimab Therapy

The linear mixed-effects model for the SCP demonstrated an overall baseline value of approximately 46% (β = 45.91, *95% CI*: 41.79–50.02, *p* < 0.001).

Time point comparisons revealed no statistically significant changes at one, two, or three months, as reflected by their respective *p*-values (>0.05). However, the SCP significantly increased by the four month (β = 2.51, *95% CI*: 0.52–4.50, *p* = 0.014), indicating a delayed but notable therapeutic effect on perfusion status. Sex emerged as a significant predictor, i.e., male participants exhibited an SCP that was 6.15 units lower than that of females (β = −6.15, *95% CI*: −11.60–−0.70, *p* = 0.027), whereas age did not appear to exert a measurable influence (β = 0.18, *95% CI*: −0.36–0.72, *p* = 0.515). These findings highlight the potential for increased perfusion in the superficial capillary plexus over a somewhat extended time while indicating a modest but statistically significant sex-related difference in SCP measurements (Figure 1).

**Figure 4:**
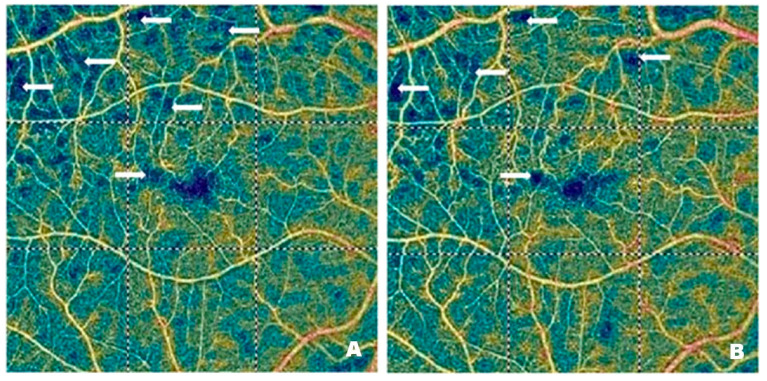
Patient 1—(**A**) Pre-treatment SCP (46.8%): blue areas of no capillary flow (arrows). (**B**) Post-treatment SCP (49.3%): slightly increased but comparable to pre-treatment; fewer blue areas (arrows).

**Figure 5:**
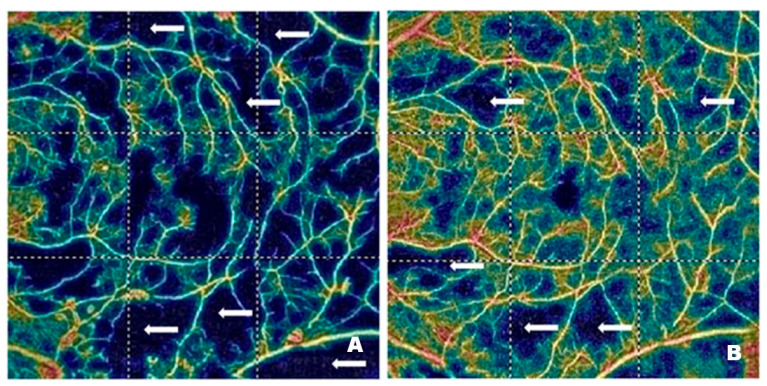
Patient 2—(**A**) Pre-treatment SCP (27.9%): extensive dark blue areas of no capillary flow (arrows); these ischemic areas are referred to as “vascular dropout” and “areas of vascular denudation”. (**B**) Post-treatment SCP (43.3%): apparent SCP improvement—significant reduction of dark blue nonperfusion areas (arrows); significant reduction of vascular dropout areas.

Figure 4 and Figure 5 demonstrate vascular network density in the SCP from two patients before (Figure 4A and Figure 5A) and after (Figure 4B and Figure 5B) treatment with intravitreal injections of faricimab. In one patient, the improvement in perfusion was slight, while in the other, it was spectacular. The blue regions marked with arrows indicate areas with reduced capillary density. The darker the blue colour, the lower the vascular perfusion, up to its complete absence (black colour). The figures, especially Figure 4A, clearly show an irregular vascular network, i.e., areas with higher and lower vessel density, areas of capillary dropout, areas of the so-called vessel pruning, and focal vessel dilations, which are largest at the border between ischemic areas and normal retina. These changes illustrate the varying density and calibre of the vessels. The reduction in blue areas shown in Figure 4B is minor, in contrast to Figure 5B, where the reduction is considerable, indicating the emergence of capillaries in the regions that were previously poorly perfused or entirely deprived of perfusion.

### 4.5. Longitudinal Evaluation of the DCP Vessel Density over Four Months of Faricimab Therapy

The linear mixed-effects model for the DCP demonstrated a clear and statistically robust increase in DCP density from baseline over the four-month therapy period. The model intercept was estimated at 39.53% (*95% CI:* 36.36–42.70, *p* < 0.001), indicating that the study population started at a moderately reduced DCP. From the first month onward, therapy-induced changes became evident, reaching a 2.54 increase (*95% CI:* 0.67–4.42, *p* = 0.008) after one month, which then steadily intensified at two months (β = 5.59, *95% CI:* 3.71–7.46, *p* < 0.001), three months (β = 8.36, *95% CI:* 6.48–10.23, *p* < 0.001), and four months (β = 9.14, *95% CI:* 7.26–11.01, *p* < 0.001). These findings suggest that the benefit to the DCP followed a progressive trajectory, with clinically meaningful improvements compounding over time.

Neither sex (β = −3.17, *95% CI:* −6.97–0.63, *p* = 0.100) nor age, centred at the median of 66 years (β = 0.06, *95% CI:* −0.32–0.44, *p* = 0.772), appeared to modify these gains significantly. Although male participants exhibited slightly lower DCP density, the confidence interval crossed zero, and the *p*-value did not support a meaningful difference between sexes. These data indicate a consistent and progressive treatment effect in older adults with diabetic macular edema, without clear evidence that demographic factors like age or sex influence outcomes (Figure 1).

**Figure 6:**
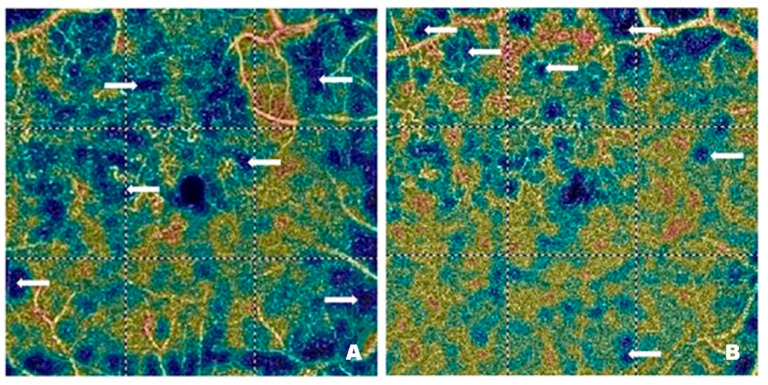
Patient 1—(**A**) Pre-treatment DCP (42.5%): areas of low capillary perfusion or vascular dropout, clearly visible as blue areas devoid of capillaries. (**B**) Post-treatment DCP (50.6%): significantly improved vascularization, increased capillary flow, significant reduction of blue areas (arrows).

Figure 6 shows vascular network density in the DCP of a patient before (Figure 6A) and after (Figure 6B) treatment with intravitreal injections of faricimab. As in the case of the SCP, the blue regions marked with arrows correspond to areas with reduced capillary density. In addition, distinct vascular patterns are visible, covering areas with varying vessel density and width. Local vessel dilations are greatest at the border between the ischemic and normal retina. Figure 6B shows a significant improvement in capillary perfusion after treatment. The vascular density map helps assess the severity of diabetic retinopathy. The improvement in retinal microcirculation after treatment, observed in both SCP and DCP, has many benefits in DR. As already mentioned, increased blood flow in the retinal microcirculation inhibits neovascularization. It also enhances the supply of oxygen and nutrients to retinal cells, thereby improving their function and preventing the degradation of photoreceptors, which are essential for vision.

### 4.6. Longitudinal Evaluation of the ORFA over Four Months of Farcical Therapy

The linear mixed-effects model for the ORFA indicated a notable decrease from baseline through the four-month follow-up. The intercept was estimated at 17.28 (*95% CI:* 14.72–19.84, *p* < 0.001), suggesting an initial ORFA increase in this patient population. From the first month onward, there was a consistent reduction in ORFA relative to baseline, reaching −1.72 (*95% CI:* −2.77–−0.66, *p* = 0.002) after one month and ultimately −3.50 (*95% CI:* −4.55–−2.45, *p* < 0.001) by the fourth month. This pattern of increasingly negative coefficients underscored a progressive, statistically robust decrease over time.

**Figure 7:**
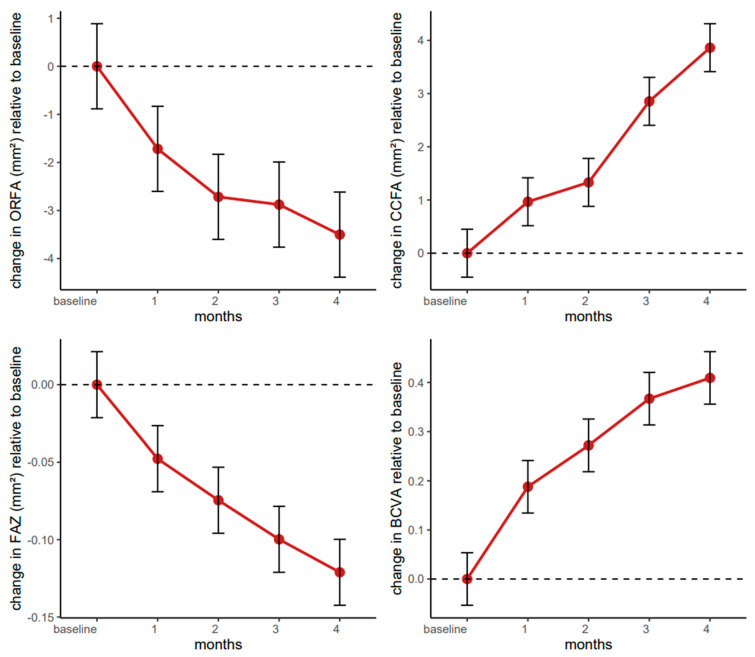
Changes in ORFA (**upper left**), CCFA (**upper right**), FAZ (**lower left**) and BCVA (**lower right**) relative to baseline over four months of faricimab therapy (the black dashed line represents the baseline level, the red solid line indicates changes in the studied parameters, and the black bars represent the 95% confidence intervals at specific time points).

Neither sex (β = −1.07, *95% CI:* −4.47–2.33, *p* = 0.533) nor age, centred at 66 years (β = 0.15, *95% CI:* −0.19–0.49, *p* = 0.380), significantly modified these changes, indicating that the observed effect was largely consistent across demographic covariates (Figure 7).

**Figure 8:**
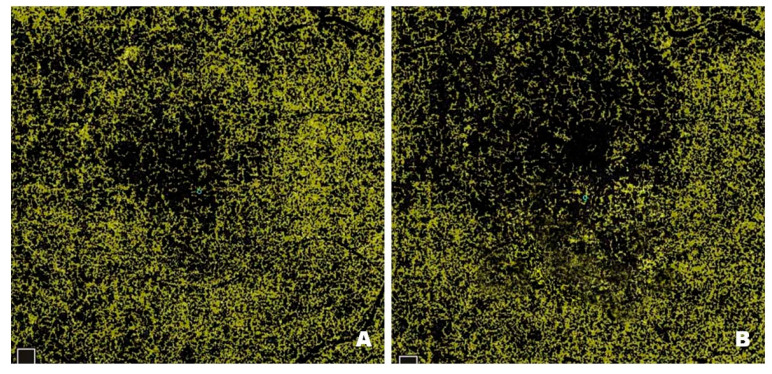
Patient 1—(**A**) Pre-treatment ORFA (18.529 mm^2^): any flow in this area (yellow) is a projection artifact or a pathology. (**B**) Post-treatment ORFA (16.959 mm^2^): enlargement of dark avascular zones, indicating the restoration of the layered structure of the retina; reduction of flow areas (yellow).

Figure 8 shows the ORFA in a patient before (Figure 8A) and after (Figure 8B) treatment with intravitreal injections of faricimab. The ORFA is a non-vascularized area of the retina; any registered flow is a pathological symptom or artefact. A reduction in any movement through this layer of the retina may indicate the effectiveness of the treatment.

### 4.7. Longitudinal Evaluation of the CCFA over Four Months of Faricimab Therapy

The linear mixed-effects model for the CCFA indicated a significant and progressively larger increase over the four-month treatment window. The estimated intercept of 22.94 (*95% CI:* 21.75–24.13, *p* < 0.001) suggested that participants had a moderately increased CCFA at baseline. Changes in CCFA showed a marginally nonsignificant increase at one month (β = 0.97, *95% CI:* −0.05–1.98, *p* = 0.062), which then reached statistical significance by the second month (β = 1.33, *95% CI:* 0.31–2.35, *p* = 0.011), with a marked increase at three months (β = 2.85, *95% CI:* 1.84–3.87, *p* < 0.001) and four months (β = 3.86, *95% CI:* 2.84–4.88, *p* < 0.001). These findings suggest a clear upward trajectory in CCFA, particularly after the first month of therapy.

Concerning covariates, sex demonstrated a statistically significant effect (β = −1.48, *95% CI:* −2.78–−0.19, *p* = 0.025); male participants exhibited slightly lower CCFA values than females. Age, centred at the median of 66 years (β = 0.04, *95% CI:* −0.09–0.17, *p* = 0.553), did not significantly modify these changes. These results highlight a notable long-term improvement in choriocapillaris flow area over successive treatment intervals, with a modest but measurable sex-related difference in baseline CCFA levels (Figure 7).

**Figure 9:**
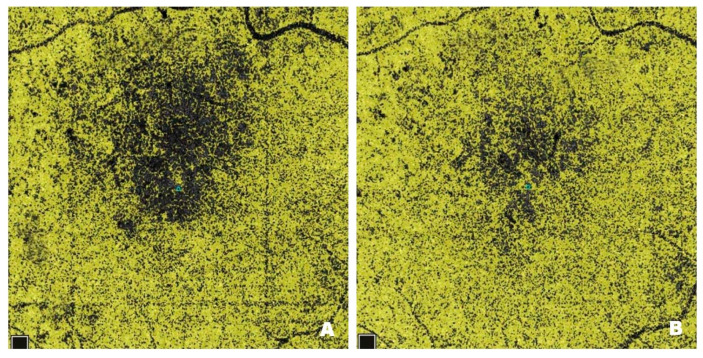
Patient 1—(**A**) Pre-treatment CCFA (28.505 mm^2^): bright, granular areas, representing vascular flow, alternating with small dark areas, probably representing intercapillary spaces; increased areas of no signal appearing as dark spots. (**B**) Post-treatment CCFA (30.580 mm^2^): reduction of non-signal areas; a more regular, less granular structure of the choriocapillaris layer.

Figure 9 shows the CCFA in a patient before (Figure 9A) and after (Figure 9B) treatment with intravitreal injections of faricimab. OCT scans reveal a granular structure of the CCFA. Bright areas represent the vascular flow, and small dark areas correspond to the intercapillary spaces. Figure 9B shows an apparent increase in blood flow through the choriocapillaris layer after treatment. There is a reduction in dark areas, and the granular structure is more regular. The choriocapillary layer transports oxygen and nutrients to the outer layers of the retina, mainly to the photoreceptors, which are the first contributors to the visual pathway.

### 4.8. Longitudinal Evaluation of the FAZ over Four Months of Faricimab Therapy

The linear mixed-effects model for the foveal avascular zone demonstrated a significant and progressive reduction from baseline through all four monthly assessments. The intercept is estimated at 0.28 (*95% CI:* 0.21–0.34, *p* < 0.001), indicating a moderate size of the FAZ area at the start. By one month, there is already a notable decrease compared to baseline (β = −0.05, *95% CI:* −0.07–−0.02, *p* < 0.001), with the reductions becoming more pronounced at two months (β = −0.07, *95% CI:* −0.10–−0.05, *p* < 0.001), three months (β = −0.10, *95% CI:* −0.13–−0.07, *p* < 0.001), and four months (β = −0.12, *95% CI:* −0.15–−0.10, *p* < 0.001). Neither sex (β = −0.01, *95% CI:* −0.09–0.08, *p* = 0.899) nor age, centred at 66 years (β = 0.00, *95% CI:* −0.01–0.01, *p* = 0.467), emerged as a significant determinant of these changes, suggesting that the observed effect is robust across different demographic covariates (Figure 7).

**Figure 10:**
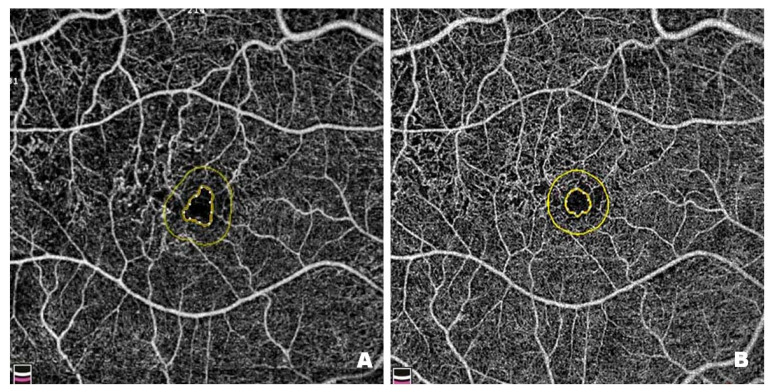
Patient 1—(**A**) Pre-treatment FAZ (0.165 mm^2^): disruption of the perifoveal capillary arcades, irregular outline and circularity loss. (**B**) Post-treatment FAZ (0.116 mm^2^): reduction in the number of disrupted perifoveal capillary arcade networks, reperfusion of perifoveal capillaries and restoration of FAZ circularity.

Figure 10 shows changes in the FAZ in a patient before (Figure 10A) and after (Figure 10B) treatment with intravitreal injections of faricimab. FAZ enlargement results from damage to the microcirculation within the macula, which is associated with retinal ischaemia. Pre-treatment FAZ is enlarged and irregular, with adjacent areas of no perfusion, disruption of the vascular arcades and widening of the perifoveal capillaries. Post-treatment scans reveal a reduction in the FAZ, restoration of its circularity, and a decrease in areas without capillary perfusion. Since enlargement and irregularities in the FAZ are among the earliest and most visible signs of DR, it is essential to monitor the size and shape of the zone in patients with this condition.

### 4.9. Longitudinal Evaluation of BCVA over Four Months of Faricimab Therapy

The fitted linear mixed-effects model for best-corrected visual acuity showed a clear and statistically significant improvement over the four-month treatment period. The estimated baseline value (intercept) was 0.38 (*95% CI:* 0.22–0.53, *p* < 0.001), indicating relatively lower acuity at the start. By one month, BCVA had already increased by 0.19 (*95% CI:* 0.13–0.25, *p* < 0.001), and this positive trend continued throughout two months (β = 0.27, *95% CI:* 0.21–0.33, *p* < 0.001), three months (β = 0.37, *95% CI:* 0.31–0.43, *p* < 0.001), and four months (β = 0.41, *95% CI:* 0.35–0.47, *p* < 0.001). Neither sex (β = −0.12, *95% CI:* −0.33–0.08, *p* = 0.243) nor age, centred at 66 years (β = −0.01, *95% CI:* −0.03–0.02, *p* = 0.604), emerged as a statistically significant covariate (Figure 7).

## 5. Summary of Faricimab Therapy Outcomes

The above results indicate that the therapeutic intervention was broadly effective across multiple morphological and functional metrics. Central retinal thickness showed robust reductions beginning from the first month. The non-perfused area did not, but it reached significance slightly later. The deep capillary plexus increased progressively with each monthly observation, whereas the superficial capillary plexus exhibited its primary significant rise by the fourth month. The outer retinal flow area decreased steadily, the choriocapillaris flow area increased significantly after the first month, and the foveal avascular zone decreased at each time point. While a few parameters (e.g., SCP, CCFA) exhibited small but significant sex-related effects, with males having marginally lower perfusion areas, age did not meaningfully alter treatment response in any of the analyses.

Overall, although the magnitude and onset of improvement differed somewhat by parameter, the treatment demonstrated a consistent pattern of benefit across both structural (e.g., CRT, FAZ) and perfusion-focused (e.g., DCP, CCFA) measures, as well as functional visual acuity (BCVA). There was no indication that either sex or older age significantly limited these therapeutic gains, supporting the view that, from a clinical standpoint, improvements were broadly sustained across different demographic covariates and ocular indices.

## 6. Discussion

Clinical studies show that 40% of patients with DME show resistance to anti-VEGF monotherapy [13,14]. Treatment response could be improved by a switch between anti-VEGF agents, intravitreal administration of corticosteroids [15] or faricimab, a bispecific angiopoietin-2 and anti-VEGF-A pathway antibody [7].

In most patients with DME, anti-VEGF therapy improves vascular perfusion. In our observation, intravitreal faricimab led to a meaningful reduction in FAZ, which was more pronounced at two, three and four months. An improvement in the FAZ circularity was also observed, which may indicate the restoration of parafoveal perfusion. Literature reports also indicate an increase in FAZ circularity within the superficial retinal vasculature after anti-VEGF therapy. Thus, the loss of FAZ circularity might be consistent with capillary atrophy at the FAZ border [10]. In addition, those who respond poorly to anti-VEGF agents have a larger FAZ in the DCP than those who respond well to treatment [16]. A larger FAZ and lower VD are seen in more severe DR types. Reduced VD at the deep capillary plexus, on the other hand, is associated with worse visual acuity, suggesting that VD may reflect the degree of capillary loss in patients with DME-related vision loss. The DCP provides about 10 to 15% of the oxygen for the retinal photoreceptors. As the DCP is the first choroid plexus to be affected in DM, assessment of changes in OCTA can help predict the evolution of visual acuity at an early stage, facilitating the monitoring and treatment of patients with DM [17].

Another parameter frequently studied in DME and discussed in the literature is the diameter of the macular vessels, which is larger compared to healthy patients. Intravitreal injections of anti-VEGF drugs have been found to reduce the diameter of these vessels, both in the SCP and in DCP, as confirmed by Massengill et al. and Kim et al. [10,18]

Ishibazawa et al. quantitatively analyzed the areas of perimacular ischemia seen in OCTA scans of patients with DM. They found that these areas were significantly larger in the SCP than in the DCP [19]. Nesper et al. measured the per cent area of non-perfusion and showed a significant increase in SCP and DCP with DR severity [20]. In DM, vessel density was reduced in both the SCP and DCP compared to non-diabetic participants [21]. Reduced VD seen on OCT angiograms, not only in the foveal and parafoveal area but also in the posterior pole, may be associated with DME. Most likely, DME affects the perfusion of the deep rather than the superficial retinal layers. The DCP is more affected by the pathological process than the SCP. It is also the site where many vascular anomalies are located [22]. The compromised perfusion of the DCP may also contribute to the development of DME because it assists the choroidal capillaries in delivering oxygen to the outer layers of the retina [23,24]. In our observation, time point comparisons of the SCP showed no statistically significant changes after the first three months (*p* > 0.05); however, after four months, a statistically significant increase in vessel density was observed (*p* = 0.014). This could be explained by the need for more extended treatment to achieve perfusion in SCP. In contrast, DCP showed a clear and statistically significant increase in vessel density throughout the four-month observation period (*p* < 0.001). There are discrepancies in the literature regarding the results of VD measurements performed before and after anti-VEGF treatment [25]. Cheong et al. observed no significant differences in vessel density in the central and parafoveal regions of the SCP and DCP after VEGF inhibitor treatment for DME compared to baseline values, nor did they observe an association of VD with retinal anatomical response to treatment. Significant damage to DCP, but not SCP integrity, was observed in patients who responded poorly to treatment compared to patients who responded well to anti-VEGF agents. The severity of outer plexiform layer (OPL) disruption in spectral-domain OCT (SD-OCT) positively correlated with the degree of DCP loss in eyes with DME [16]. In contrast, Massengill et al. showed that eyes with improved BCVA showed reduced vessel diameter at the SCP level, while eyes with reduced central subfield thickness showed increased perfusion density and vessel length density in the DCP [10].

One theory for the improvement in macular perfusion in DCP, once macular edema has subsided, is that it is due to the reduction of intraretinal cystoid spaces in the fovea and the resulting re-entry of previously displaced capillaries into their original location [26].

NPAs constitute a significant problem in DR pathophysiology of DR involves biochemical and structural changes in the retinal blood vessels, leading to capillary atrophy and, consequently, areas of retinal ischemia and the formation of NPAs, which in turn can result in the development of neovascularization. There are conflicting data in the literature regarding the effects of anti-VEGF injections, regardless of the type of molecule used. However, VEGF is considered a significant factor in the pathogenesis of retinal ischemia in patients with DM; therefore, intravitreal anti-VEGF treatment may reduce NPAs. In our observation, faricimab treatment resulted in a decrease in NPAs, particularly evident from the second month onward, reaching high statistical significance (*p* < 0.0001). It was also observed that NPAs were more extensive in men, while age had no significant effect on NPA size. Potential mechanisms explaining reperfusion after anti-VEGF treatment include restoration of the normal anatomical structure of the retina and remodeling of the retinal microcirculation due to regeneration of pericytes and basement membranes. VEGF suppression can also reduce leukostasis and allow small vessels to reopen. Different pathophysiologies of macular ischemia and peripheral ischemia may account for the opposite results regarding reperfusion of NPAs in the macula and retinal periphery observed after anti-VEGF treatment.Macular ischemia has been attributed to pericyte loss, excessive vascular wall permeability and VEGF upregulation, while peripheral ischemia is mainly due to the thickening of the basement membrane of the vessel walls. However, the exact mechanism has not yet been clarified [27]. However, Levin et al. observed post-treatment reperfusion of peripheral areas in 75% of eyes with DME and PDR, which previously lacked capillary perfusion. The treatment consisted of at least one intravitreal injection. Peripheral reperfusion of ischemic areas of the retina was assessed on ultra-widefield fluorescein angiography, performed within 5 months of the last intravitreal anti-VEGF injection in the study eye [28]. Some studies reported no significant change in macular ischemic areas after anti-VEGF treatment in patients with DME. There are also studies describing a worsening of macular ischemia after anti-VEGF therapy in patients with DME or PDR. Therefore, it has been suggested that the dose regimen of anti-VEGF agents may also affect the retinal perfusion status [27].

## 7. Conclusions

In patients with DME, intravitreal injections of faricimab, an anti-VEGF and angiopoietin-2 inhibitor, improved retinal microvascular parameters over a four-month observation period, as shown on OCT angiograms. A reduction in the FAZ, an increase in FAZ circularity, and an increase in VD at the DCP level were found. The SCP vessel density increased after the fourth month. A reduction in NPA size and increased CCFA flow were also observed. Vessel density in the ORFA decreased, indicating a tendency to restore the normal nonvascular structure of this area. The effect was most pronounced in eyes with BCVA improvement and CRT reduction; the two parameters were negatively correlated.

It should be emphasized that there is no uniform, state-of-the-art software package for the analysis of OCT angiograms. Research studies are based on different analysis algorithms; discrepancies in the results should, therefore, be kept in mind. A more detailed analysis is needed so that patients strongly responding to anti-VEGF drugs can be analyzed separately from those exhibiting poor responses. This would allow for a more accurate understanding of the effects of anti-VEGF agents on retinal blood vessels in DR.

## Data Availability

The original contributions presented in this study are included in the article.
